# Micro-Mechanical Investigation of Interfacial Debonding in Carbon Fiber-Reinforced Composites Using Extended Finite Element Method (XFEM) Approach

**DOI:** 10.3390/mi13081226

**Published:** 2022-07-30

**Authors:** Raees Fida Swati, Saad Riffat Qureshi, Muhammad Umer Sohail, Adnan Munir, Omer Masood Qureshi, Abid Ali Khan

**Affiliations:** 1 Department of Aeronautics & Astronautics, Institute of Space Technology, Islamabad 44000, Pakistan; srqureshi@mail.ist.edu.pk (S.R.Q.); umer.sohail@mail.ist.edu.pk (M.U.S.); 2 School of Mechanical & Manufacturing Engineering (SMME), National University of Sciences and Technology (NUST), Islamabad 44000, Pakistan; adnan.munir@smme.nust.edu.pk; 3 Automotive Design and Crashworthiness Research, Islamabad 44000, Pakistan; omq@adcr.com.pk; 4 Aeronautical Engineering Department, Military Technological College, Muscat P.O. Box 262, Oman; abid.khan@mtc.edu.cn

**Keywords:** micro-crack model, interfacial micro-debonding, carbon fiber-reinforced composites, extended finite element method (XFEM)

## Abstract

The interface debonding in carbon fiber-reinforced polymers is analyzed and evaluated using the extended finite element method (XFEM). In order to accurately evaluate the bonding properties between fibers and matrix, different tests were carried out, including the multiple tests for different orientations to study longitudinal, transversal, and shear properties of unidirectional carbon fiber-reinforced composites. Extensive experimentation has been performed in all the different groups and categories with different dimensions and parameters in order to ascertain the values of strength and the prediction of the damage to the structure. The experimental and numerical comparison provided significant trends and data to evaluate the mechanical properties of the interface. The values of stiffness and strength are compared and validated. Development of Representative Volume Element (RVE) for progressive damage model to these damage phenomena has already been performed as a feasibility study for the model, though it is not included in this particular paper. The results of this research for all the experimental and numerical sets can serve as reliable data in the microsimulation of devices and sensitive parameters that include carbon fiber-reinforced light metal matrix composites and makes a better investigative model that contributes to various conditions. It further offers an investigation of the microscopic deformation mechanisms in the composites.

## 1. Introduction

The emergence of a new class of fiber composite materials for use in aircraft has its origin in a number of technical and scientific developments, the starting point being the discovery last century of synthetic organic materials derived from vegetation and coal. The development of carbon fibers of high strength and stiffness creates special problems because the graphite crystal from which they are produced is extremely anisotropic. The crystals have to be aligned with the fiber axis to obtain high performance. Fiber composites are making an impact across the whole field of structural materials and their use has been growing at about 7% per annum for the last decade and a half, regardless of the economic downturns. It is, however, the requirements of high-performance aircraft for defense and civil usage, aerospace vehicles, and rockets that have fueled the growth in the development of fiber composites. The straightforward requirement for weight loss favors their use [1,2,3].

The fiber–matrix interface is a critically thin surface between fiber and matrix. A key role is placed by the interface in determining the mechanical properties of composite materials because the load applied externally has to be transmitted from matrix to fibers through the interface [4,5,6]. The bonding quality between the fibers and matrix is responsible for the strength of the fiber–matrix interface. Bonding at the fiber–matrix interface is particular for every system. A complex chemical bond between fiber and matrix is formed in the case of the carbon/epoxy composites. Composites having a strong interface have high strength and stiffness as compared to composites having a weak interface. Composites having a strong interface behave in a brittle manner with 2–3% elastic elongation-to-failure with no sufficient plastic deformation. Therefore, in some special cases, a relatively weak interface is required. The most commonly observed failure in composites is delamination. It is the separation of layers due to the weakness of the interface between laminates. Delamination may originate from low-velocity impact, strangeness in structural load paths that cause out-of-plane loads, or from heterogeneous and stacked nature, which create local out-of-plane load [7,8,9].

The development of Representative Volume Element RVE [10,11,12] will provide us with more strength and make our research even more interesting as our domain of research is wider now. In any research, it is very important to know what the previous theories, hypotheses, or findings of researchers and scientists are. When we can understand such previous work and research, we can then take it forward to make it even better. The main portion of usage of carbon fiber-reinforced composites is in the aerospace industry including military operations, space operations, and civil aircraft, where weight reduction is critical for higher speeds and increased payload [13,14,15]. In aerospace, the use of fiber-reinforced polymers has experienced steady growth. The structural integrity and durability of these early components have built up confidence in their performance and promoted developments of other structural aircraft components, resulting in an increasing number of composites being used in military aircraft. For example, the airframe of an AV-8B contains 25% by weight of carbon fiber-reinforced epoxy, while the F22 contains 25% by weight of carbon fiber-reinforced polymers. The stealth characteristics of these aircraft are because of the usage of carbon fiber-reinforced composites. In commercial aircraft, these composites began to be used along with a few secondary components. The Boeing 777 contains carbon fiber-reinforced epoxy 10% by weight which was introduced in 1995. On the other hand, the Boeing 787 Dreamliner, which was introduced in 2009, contains carbon fiber-reinforced polymers of almost 50% by weight [16].

Damage progression includes three main technology areas for obtaining a solution: instrumentation and data processing hardware, data integration, and predictive modeling. It is believed that damage progression is, in most cases, an emerging technology. The general modeling issues that we consider are service environment, considerations in material specification, and material microstructure considerations (including single phase materials and multiphase materials). First, we start with forms of damage that can occur in our selected type of material, which can be found in many forms within the same material. The generic working model we used here is any structural deviation from a perfect material state, such as material separations, imperfections, and displacement discontinuities. Damage occurs virtually at all length scales which results in non-linear, irreversible dissipative deformation mechanisms manifesting as history-dependent behavior. Usually, damage occurs at lower length scales, evolves and interacts with these scales, and then manifests with higher scales. As damages occur at the microscale, modeling relative volume elements to begin with will provide us with more knowledge on how that damage propagates in any material. Thereafter, by characterizing that material’s behavior it will then be useful to consider a monotonic stress–strain curve which shows material behavior from initial loading up to material failure. After generating stress–strain curves, we can predict the total life or the remaining life of the material [4].

## 2. Material Models

Material properties of chosen fiber-reinforced composites are given in Table 1, Table 2 and Table 3 which are standard properties. The homogenized values are also calculated for the interfacial debonding study.

Here ***Em*** represents elastic modulus and ***υ*** as poisson ratios whereas X_T_ and X_c_ are tensile and compressive strengths, respectively.

## 3. Experimental Study

### 3.1. Experimental Standards and Specimen

**Figure 1:**
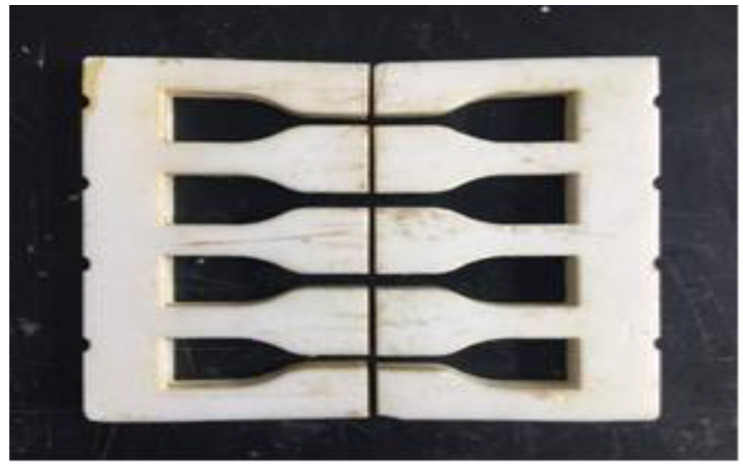
Tensile sample die.

Carbon Fiber-Reinforced Composites (CFRCs) are modeled for the conditions of the multiscale XFEM framework, followed by the development of an enrichment scheme of equations. The proposed micro-mechanical model for the three-point bending test is validated by comparison with experimental data. The study provided a significant benchmark for the prediction of the XFEM progressive damage [14,18,19] and a framework for the mechanical behavior of carbon fiber-reinforced composites. A three-point bending test is performed to determine the bending stress, flexural stress, and the flexural strain in the composite materials. The three-point bending test is carried out in the same machine described previously, the test samples are prepared according to ASTM standards. The dimension design of transverse fiber tow tensile specimens refers to the standard sample size recommended by ASTM D638, as shown in Figure 1 processed through Northwestern Polytechnical University, China. The die is cut along the symmetrical middle line of the resin casting die for laying the fiber tows. In order to facilitate demolding, the following setup is used as a die mechanism in this experiment.

**Figure 2:**
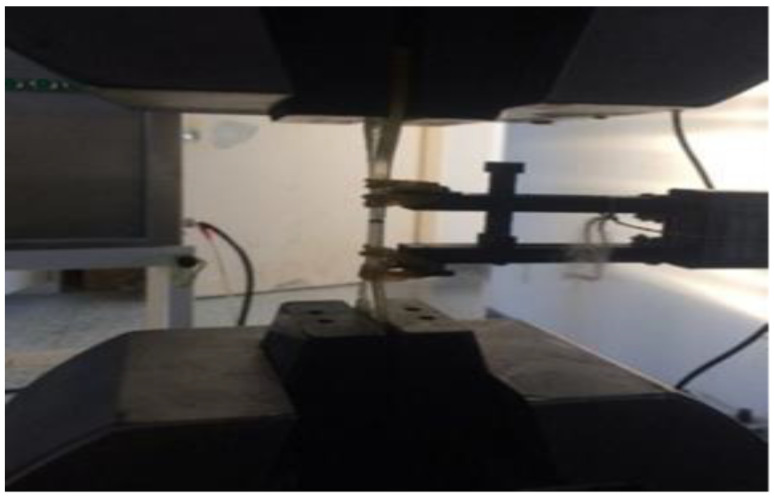
Tensile sample die.

The experiment adopts a WDW-100 micro-controlled electronic universal testing machine as shown in Figure 2, processed through Northwestern Polytechnical University, China. The engineering constants of epoxy and carbon fiber are already discussed. The accuracy of load reading should be higher than 1% of the measured value. The fixture system can ensure that the center line of the sample is consistent with the center axis of the testing machine. The tension tester is equipped with an extensometer, which is connected to a continuous recording device to automatically record the elongation of the specimen in the clamp. The testing process refers to ASTM 3039. processed through Northwestern Polytechnical University, China.

Understanding the complicated damage mechanism of composites is still challenging. With the widespread use of CFRP, research into the damage mechanisms of CFRP structures to CFRP development in engineering structures is of major concern. Latest improvements focus on the interlaminar interface in modeling delamination propagation. Multi-layered delamination [8,20] is usually the result of damage in composite laminates where it grows up and transforms into numerous interlaminar interfaces.

### 3.2. Experimental Investigation of Interfacial Bonding and Mechanical Properties

**Figure 3:**
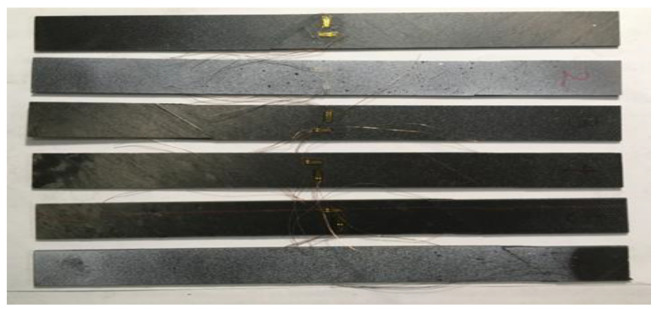
Samples.

**Figure 4:**
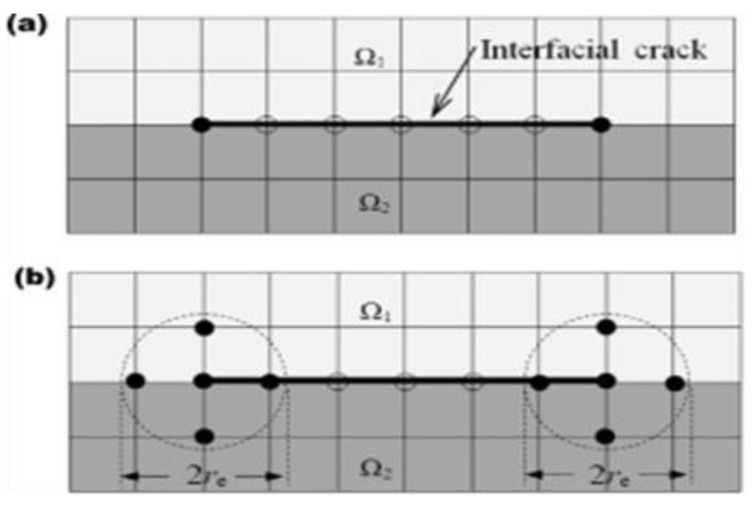
Tension specimen for experimentation (**a**) domain and (**b**) estimation for radii analysis. A half of FE mesh was used.

To accurately evaluate the bonding properties between fibers and matrix, the tensile tests were carried out, including the tensile tests of transverse fiber orientation and the shear tests of 45-degree fiber orientations. Figure 3 shows the samples ready for the test and Figure 4a,b represents the scheme for an interfacial crack within the domains Ω_1_ and Ω_2_ for the corresponding radii r_e_.

The mechanical properties of unidirectional lamina and adhesive are presented in Table 4. Considering the symmetric and boundary conditions of the panel, within the model.

### 3.3. Testing of Transverse Bond Strength at Interface for CFRCs

**Figure 5:**
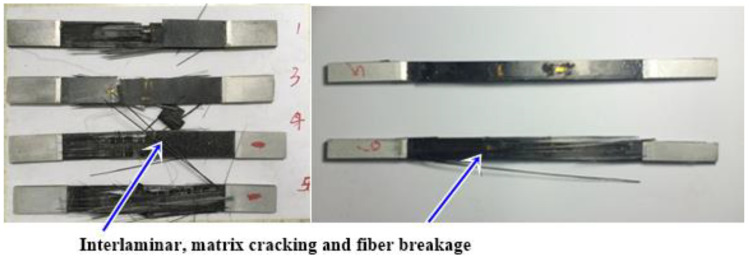
Test results of the different specimen.

In the middle of the resin stretching spline die, a slot is opened, the fiber tow is laid perpendicular to the direction of the sample stretching, and the transverse tow stretching sample prepared by co-curing after pouring resin is stretched to fracture under uniform loading of the appropriate mechanical device [21]. Tensile strength, modulus of elasticity, and strain at maximum load are calculated according to the tensile stress–strain curve. Previous studies have shown that the tensile strength of transverse tow tensile specimens can reflect the strength of the fiber/resin–interface bonding. The transversal damage has been particularly studied in one of the recent studies of the author. The tested specimens are shown in Figure 5.

### 3.4. Interfacial Shear Strength Test of CFRPs

**Figure 6:**
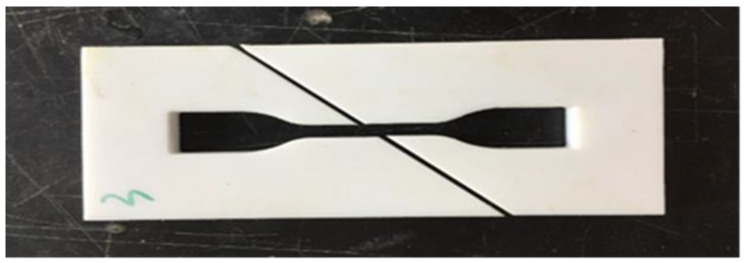
Shear strength testing die for fiber arrangement.

**Figure 7:**
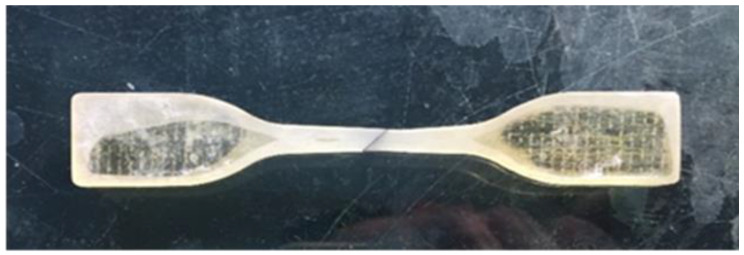
Grinding qualified resin matrix spline.

The interfacial shear tow tension specimen prepared by co-curing after pouring resin is stretched to fracture under uniform loading of the appropriate mechanical device. According to the tensile stress–strain curve, the interfacial shear strength and shear elastic modulus are calculated. Dimension design of tensile specimens of oblique 450 fiber arrangements refers to the standard sample size recommended by ASTM D638 [22] processed through Northwestern Polytechnical University, China. Along the symmetrical center line of the resin casting die, the die is cut diagonally 450 to lay the fiber arrangements. The other methods of operation are the same as those of transverse fiber tow tensile specimens. The drawing sample die is shown in Figure 6. The final curing and polishing of qualified resin matrix splines are shown in Figure 7.

## 4. Numerical Model Estimation and Representation

The dimensional relations among its different components were still undetermined. We have one full cylindrical fiber at the center and four quarter cylindrical fibers at all corners for the sake of periodicity. So, we have a total of two carbon fibers other than carbon. Now we can say that the total volume occupied by the carbon fibers is equal to the volume of the unit cell multiplied by the fiber volume fraction which is 0.6. This is shown in Equation (1), where $V_{f}$ and $V_{c}$ represent the volume of carbon fibers and the cubical unit cell. Equation (2) simplifies the expression of the volume of fiber and the unit cell. Where “a” represents the length of the sides of the unit cell and $“r_{f}”$ represents the radius of the carbon fiber. Here, VFF is the volume filler fraction.

(1) $$2\left(V_{f}\right)=VFF\;\left(V_{c}\right)$$

(2) $$2\left(\pi \times r_{f}{}^{2}\right)\times a=VFF\;\left(a^{3}\right)$$

The relationship is presented in Equation (3), which gives us the length of the unit cell.

(3) a=2π×rf2VFF

Using the same concept as the above equations, we derived the expressions given in Equations (4) and (5). The volume fraction for the carbon fibers was selected to be 0.5% of the total matrix volume which is less value compared to the carbon fibers and it will still give satisfactory results for stress-bearing in the normal direction to the carbon fibers.

(4) $$V_{nf}=0.005\times V_{m}$$

(5) $$V_{snf}=\pi {\left(r_{nf}\right)}^{2}\times a$$

(6) $$N_{n}=V_{nf}/V_{snf}$$

where $V_{nf}$ and $V_{snf}$ are the total volume of Nano-fibers and the volume of a single Nano-fiber, respectively. We can calculate the number of Nano-fibers in our unit cell by using the expression given in Equation (6). The $r_{nf}$ represents the radius we selected for our Nano-fibers. Using all of the above expressions, we calculated different dimensions of our representative volume element, which are given in Table 5. We calculated the number of Nano-fibers to be 14 and, as already discussed, we have two carbon fibers. We selected our fiber diameter to be 9 µm which is the most common diameter of the Carbon T-300 fiber, processed through Northwestern Polytechnical University, China being used these days.

The Extended Finite Element Method (XFEM) is based on the Finite Element Method (FEM) used to treat discontinuities. The objective of the research is to evaluate the bonding properties between fiber and matrix and to define a technique for comparison of mechanical properties i.e., strength and stiffness in interfacial debonding. This will help us improve the interfacial properties of composite laminates in order to avoid delamination. Implementation of XFEM [23,24,25] and simulations of different loading conditions for the proposed ECDM model and the modified CZM are tools for the prediction of the crack growth due to delamination. The simplification of the modeling of discontinuous phenomena is the main benefit of XFEM methods for different problems in materials science. In the traditional formulation of the FEM, a fracture is modeled by requiring the fracture to follow element edges. In contrast, the fracture geometry in the X-FEM does not need to be aligned with the element edges, which is a great flexibility.

In damage analysis, there are, in general, two different types of failure criteria for CFRCs material. Strength of Material Criteria based on stresses for the damage initiation and Fracture Mechanics Criteria based on energy for the damage growth. Hashin and Rotem (1973), Hashin (1980), Matzenmiller et al. (1995), and Camanho and Davila (2002) all contributed to the Abaqus anisotropic damage model. Fiber rupture in stress, fiber buckling and kinking in compression, matrix cracking under transverse tension and shearing, and matrix crushing under transverse compression and shearing are all taken into account. In Abaqus, the initiation criteria proposed by Hashin and Rotem (1973) and Hashin (1980), in which the failure surface is expressed in the effective stress space, determine the onset of damage. The response of the material is computed from Equation (7).

(7) $$\sigma =C_{d}\varepsilon$$

In Equation (7) *ε* is the strain and *C_d_* is the elasticity matrix, which reflects any damage and has the form shown in Equation (8).

(8) $$C_{d}=\frac{1}{D}\left[\begin{array}{ccc} \left(1-d_{f}\right)E_{11} & \left(1-d_{f}\right)\left(1-d_{m}\right)v_{21}E_{11} & 0\\ \left(1-d_{f}\right)\left(1-d_{m}\right)v_{12}E_{22} & \left(1-d_{m}\right)E_{22} & 0\\ 0 & 0 & \left(1-d_{s}\right)G_{12}D \end{array}\right]$$

**Figure 8:**
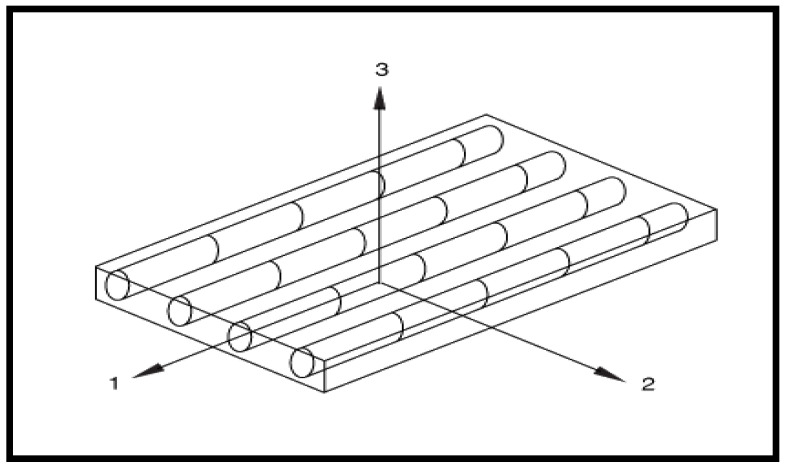
Unidirectional CFRC lamina.

In Equation (8) $D=1-\left(1-d_{f}\right)\left(1-d_{m}\right)v_{12}v_{21}$, $d_{f}$ is the current state of fiber damage, $d_{m}$ is the current state of matrix damage, $d_{s}$ is the current state of shear damage, $E_{11}$ is the Young’s modulus in fiber direction (longitudinal direction), $E_{22}$ is the Young’s modulus in the direction perpendicular to the fibers (transverse direction), *G*_12_ is the shear Modulus and $v_{12}$, and $v_{21}$ are Poisson ratios. The longitudinal and transverse direction of uni-directional lamina is shown in Figure 8 as direction 1 and 2, respectively.

The damage variables $d_{f}$, $d_{m}$, and $d_{s}$ were derived, from the damage variables $d_{f}^{t}$, $d_{f}^{c}$, $d_{m\;}^{t}$, and $d_{m}^{c}$ shown in Equations (9)–(13) which were referred to in the four failure modes discussed previously.

(9) $$d_{f}=\{d_{f\;}^{t}\;if\;\sigma_{11}\geq 0$$

(10) $$d_{f}=\{\;d_{f\;}^{c}\;if\;\sigma_{11}<0$$

(11) $$d_{m}=\{\;d_{m\;}^{t}\;if\;\sigma_{22}\geq 0$$

(12) $$d_{m}=\{\;d_{m\;}^{c}\;if\;\sigma_{22}<0$$

(13) $$d_{s}=1-\left(1-d_{f\;}^{t}\right)\left(1-d_{f\;}^{c}\right)\left(1-d_{m\;}^{t}\right)\left(1-d_{m\;}^{c}\right)$$

The damage variables $d_{f\;}^{t}$,$\;d_{f\;}^{c}$,$\;d_{m\;}^{t}$, and $d_{m\;}^{c}$ in Equations (9)–(13) are the internal damage variables in the fiber and matrix phases of the lamina, under tension or compression loadings. Damage initiation refers to the beginning of a degradation. For CFRCs, it is based on Hashin’s failure criteria, using four different damage mechanisms that is fiber tension, fiber compression, matrix tension, and matrix compression as shown in Equations (14)–(17).

Fiber Tension: $\left(\sigma_{11}\geq 0\right)$

(14) $$F_{fT}={\left(\frac{\sigma_{11}}{X_{1T}}\right)}^{2}+\alpha {\left(\frac{\tau_{12}}{S_{12}}\right)}^{2}=1$$

Fiber Compression: $\left(\sigma_{11}<0\right)$

(15) $$F_{fC}={\left(\frac{\sigma_{11}}{X_{1C}}\right)}^{2}=1$$

Matrix Tension: $\left(\sigma_{22}\geq 0\right)$

(16) $$F_{mT}={\left(\frac{\sigma_{22}}{X_{2T}}\right)}^{2}+\alpha {\left(\frac{\tau_{12}}{S_{12}}\right)}^{2}=1$$

Matrix Compression: $\left(\sigma_{22}<0\right)$

(17) $$F_{mC}={\left(\frac{\sigma_{22}}{2S_{13}}\right)}^{2}+\left[{\left(\frac{X_{2C}}{2S_{13}}\right)}^{2}-1\right]\left(\frac{\sigma_{22}}{X_{2C}}\right)+{\left(\frac{\tau_{12}}{S_{12}}\right)}^{2}=1$$

In Equations (14)–(17) $\sigma_{11}$,$\;\sigma_{22}$, and $\tau_{12}$ are the longitudinal, transverse, and shear stresses in the lamina, $X_{1T}$ and $X_{1C}$ refers to the tensile and compression strength in the fiber direction (longitudinal tensile and compressive strength), and $X_{2T}\;$ and $X_{2C}\;$ refers to the tensile and compression strength in the transverse direction. $S_{12}$ and $S_{13}$ are the longitudinal and transverse shear strength, respectively. The coefficient α determines the contribution of shear stress to the fiber tensile damage initiation in the present work. The material was linearly elastic before damage initiation, based on the brittle behavior of the CFRC. Damage evolution took place after the damage was initiated. After one or more damage initiation conditions are met, the damage evolution description describes how the material degrades. Multiple damage evolution forms, one for each given damage initiation criterion, can act on a material at the same time. Damage variables that have values ranging from zero (undamaged state) to one (damaged state) influence the reduction of stiffness coefficients. For the Hashin’s damage evolution model, the data table contains the fields shown in Table 6 and a brief comparison in Table 7 with statistical segregation. You define damage stabilization for fiber-reinforced materials by entering viscosity coefficients for each of the potential failure modes. Each of the viscous coefficients should be small compared to the increment size. Viscous regularization is intended to improve convergence as the material fails.

Whilst the matrix crack propagated across the fiber–matrix interface, it also deflects alongside the interface. Fracture mechanics and shear strength methods are the best ways to deal with the interface debond problem. The fracture mechanics method deals the fiber–matrix interface debonding as a crack propagation problem, in which the interface debonding [8,26] happens because the strain energy release rate at interface reaches debonding toughness. Moreover, the shear strength method is based on maximum shear stress criteria. Sun and Singh analyzed matrix multi-cracking and fiber/matrix interfacial debonding. However, for an extended interface debond period, the fracture mechanics approach provided a good fit. The fracture mechanics approach is used to determine the fiber/matrix–interface debonding, and is given as: Applied the XFEM step enrichment to model narrow damage localization zones as given in Equation (18) for interface debond energy and Equation (19) for the interface debond length. Table 6 shows the numerical evaluation and parameter of selection for failure in different scenarios.

(18) $$\zeta d=\frac{F}{4\pi rf}\frac{\partial wf\left(0\right)}{\partial Ld}-\frac{1}{2}\int_{0}^{Ld}\tau i\frac{\partial v\left(x\right)}{\partial Ld}dx$$

where *ζ_d_* is interface debond energy, *F* is fiber load at matrix crack plane, *v*(*x*) is relative displacement between fiber and matrix, and $w_{f}$(0) shows the fiber axial displacement at the matrix crack plane. The interface debond length $L_{d}$ is determined by equation.

(19) $$L_{d}=\frac{r_{f}}{2}\left(\frac{V_{m}E_{m}\sigma }{V_{f}E_{c}\tau_{i}}-\frac{1}{\rho }\right)\;-\sqrt{{\left(\frac{r_{f}}{2\rho }\right)}^{2}+\frac{r_{f}V_{m}E_{m}E_{f}}{\tau_{i}^{2}E_{c}}}\zeta_{d}$$

Table 8 shows the predicted values for the model and comparison with the literature and previous studies with the shear dominated failure criterion in Table 6 and Table 7, for all the variables and properties previously discussed. The result’s predicted values lie in a very good agreement for longitudinal strength prediction. It clearly represents the validation for the transversal and longitudinal principle for the unidirectional fibers. Where $X_{T}$ are tensile, and $X_{C}$ are compressive strengths for the set.

**Figure 9:**
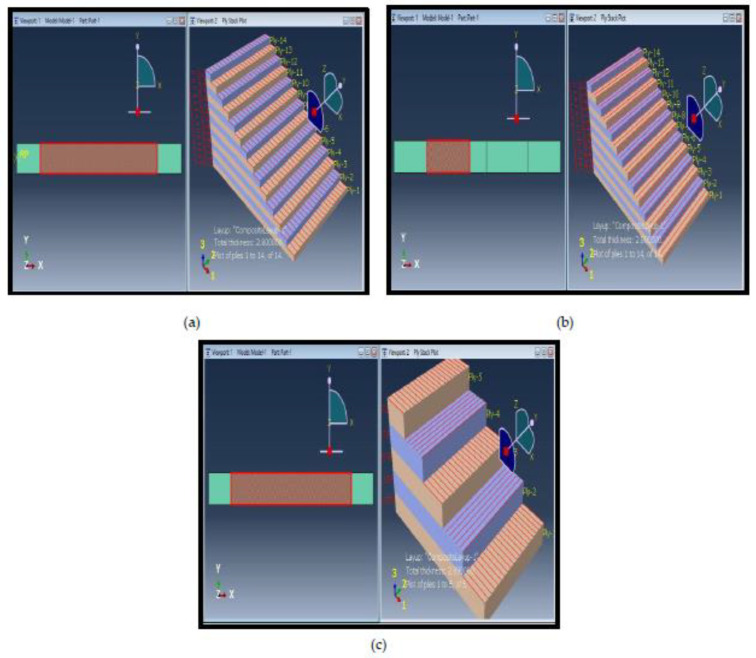
Model geometry of tensile specimen: (**a**) category A, (**b**) category B, and (**c**) category C.

A 3D model is developed with geometry dimensions similar to experimental specimens which is shown in Table 4, Table 5 and Table 6. In comparison to natural specimen geometry, the geometry of the numerical model is made to appear as authentic as possible. A three-dimensional, deformable shell planar function is used to build the model. Figure 9 shows that three specimen geometry with different orientation. 7a shows the zero-degree fiber orientation and 14 plies, 9b shows the 90-degree fiber orientation with 14 plies, and 9c shows 5 plies with 0/90/0/90/0 plies orientation.

The research started with tensile testing to study the transversal damage for category A, B and C [27]. Specimen A and B are composite layups of 0° and 90°, respectively, whereas Specimen C is a cross-ply composite layup of 5 plies. One ply usually consists of two constituents, fiber and matrix, which can both be damaged individually. A laminate is made by piling multiple plies together in different orientations. The lay-up is the arrangement of the laminate that shows its ply composition with various fiber orientations. The detailed model and the specimen are shown in Table 8, Table 9 and Table 10 and the representation is shown in Figure 9a–c.

## 5. Major Critical Results and Discussions

**Figure 10:**
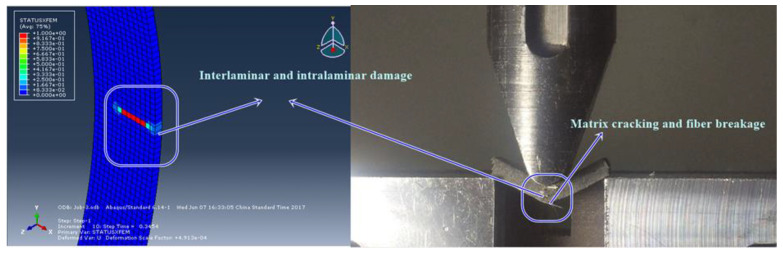
XFEM damage comparison for 3P/0° specimen.

**Figure 11:**
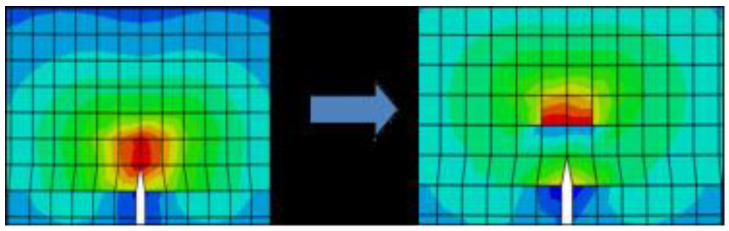
Crack propagation in vertical direction.

**Figure 12:**
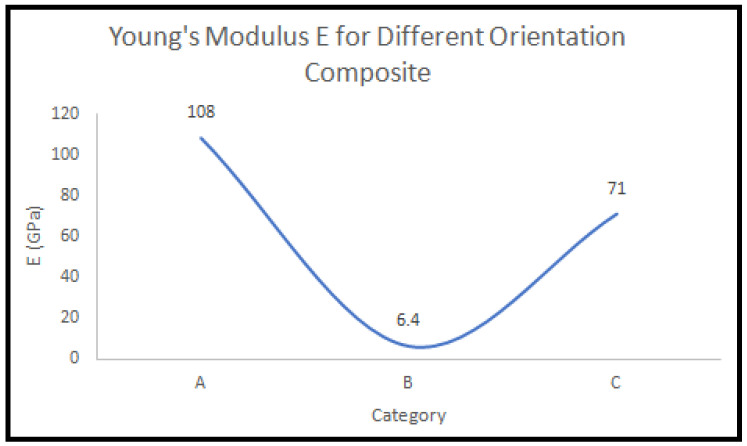
Modulus of elasticity of categories.

Furthermore, it is followed by the comparison of stress component to the fiber (vertical) direction for reference models on beginning the crack propagation for a similar width and the simulations are shown below in Figure 10 for XFEM damage comparison for 3P/ 0˚ specimen which depicts a very reliable model in Figure 11 and Figure 12. The experimental method is the same as that of transverse fiber tow tension. XFEM damage specimen. The same is crystal clear from Table 11, Table 12 and Table 13 for the comparison of models for the stresses and overall strength, which is promising.

The experimental results in Figure 10 and Figure 11 display the damage and crack propagation as comparison and related load–displacement curve and contour plots for every carbon fiber composite ply as the crack and delamination. It is clearly proven that the transversal damage for the group A of 45 has crack initiation at the center of the specimen, which can be evaluated from the other cases. After the failure of the specimen, from load and displacement data points and analyzes the results.

The curves clearly illustrate the anisotropic brittle behavior of carbon-reinforced composites. When a load is applied in fiber direction (longitudinal direction) Figure 10 clearly illustrates that aligned continuous fibers have a much higher tensile strength.

It is possible to obtain the mechanical properties of composite material after conducting tensile tests and processing the results, as shown in Table 14. It is important that the material has high mechanical strength and strain values, and that the elastic modulus is in the right range, a representation for a sorted set of experiment is shown in Figure 12.

**Figure 13:**
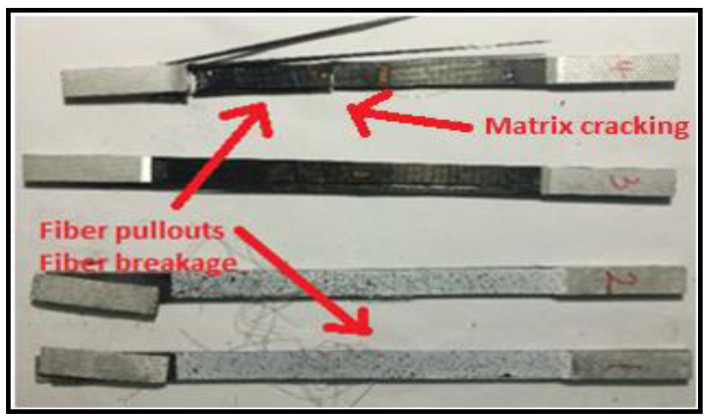
Matrix and fiber breaking.

Different fracture modes, such as brittle matrix fracture and fiber splitting, were also observed in the test results as shown in Figure 13.

The initiated crack propagates in the upper surface that causes the initiation of damage to the adjoining layers and comparative studies have been considered as [28], the result lies in a reasonable agreement. The values had been calculated as a comparison of the predicted values and the results from the experimental outputs had been numerically calculated as given in Table 14.

The boundary conditions and the displacement load for 90-degree orientation have been analyzed and lies within the error range of less than 3%. It is observed that in the case of transverse tension or compression, the phenomenon of transverse bearing capacity of the matrix loses when the overall bearing capacity of the matrix is damaged.

At the end of the test, if the fiber bundles are intact and only the resin at the standard interval necks or breaks, which belongs to the invalid mode, the test results will be discarded. Table 15 shows the detailed results.

## 6. Conclusions

This experimental work is in agreement with numerical simulations which provides deep understanding of crack behavior in carbon fiber-reinforced composites, particularly for the interfacial micro-debonding. The extensive experimentation and numerical analysis has been carried out for the development of a flexible framework for the prediction of the damage computational model, techniques for fracture and delamination behaviour of the crack in carbon fiber-reinforced composites, particularly for the different modes of loads, and boundary conditions have been evaluated using XFEM. The experimentation and numerical response of the framework is in a good agreement, hence it conforms to the proposed technique as a simplified and efficient tool for beginners and professionals. This comparative study has also been carried out with previous numerically studied sets and the results lie in reasonable convergence. The results obtained with this technique are in agreement with experimental results and the error percentage is quite reasonable. This research has a unique methodology by considering different phenomenon at the same time for interfacial debonding strength. It is recommended to carry out further quantitative work in order to validate the numerical algorithms. The stress–strain behavior gives the reader a better perspective in order to investigate various affects on the composite. This study is concise and converged with the previously performed models and research for all sets of results which were grouped into multiple categories. This study has been carried out for both transversal damage and the phenomenon of delamination in composites with the multiple modes and categories A, B and C.

## Figures and Tables

**Table 1:** Material properties and homogenized values.

Linear	*Em* (GPa)	*υ*_*m*_	*υ*_*p*_
Elasticity	3.73 ± 0.30	0.38 ± 0.01	0.30
Damage	*σft* (MPa)	*σft* (MPa)	*GIC* (J/m^2^)
Model	61.6 ± 4.6	300 ± 30.6	334.1 ± 73

**Table 2:** Material properties and homogenized values for the development of RVE.

Material	Elastic Modulus (GPa)	Poisson’s Ratio	Density (kg/m^3^)
**Carbon Fiber** **(T-300)**	231	0.2	1760
**Matrix** **(Cast Epoxy)**	3.425	0.32	1250

**Table 3:** Mechanical properties and parameters for the unidirectional set for the interfacial debonding model [17].

	E_11_ (MPa)	E_22_ (MPa)	G_12_ (MPa)	G_23_ (MPa)	*υ*_12_	X_T_ (MPa)	X_C_ (MPa)	S
Carbon Fiber	221	13.8	9	4.8	0.2	3528	2500	-
Epoxy Resin	3.5	3.5	1.296	1.296	0.35	112	241	89.6

**Table 4:** Mechanical properties of carbon fiber in analysis.

*E*_11_	*E*_22_ = *E*_33_	*G*_12_ = *G*_13_	*G*_23_	*v*_12_
245 (GPa)	19.8 (GPa)	29.191 (GPa)	5.922 (GPa)	0.28

**Table 5:** Different dimensions of RVE.

Parameters	Symbol	Value	Units
**Fiber Radius**	$r_{f}$	4.5	µm
**RVE Length**	a	14.5621	µm
**Nano-Fiber Radius**	$r_{nf}$	0.1	µm

**Table 6:** Shear dominated failure assessment.

Shear Dominated Failure	NU-Daniel	Applied Criteria [14]
$\left(\sigma_{22}^{Tran}≺\sigma_{22}\leq 0\right)$	${\left(\frac{\tau_{12}}{S^{L}}\right)}^{2}+\frac{2}{\alpha }\frac{\sigma_{22}}{S^{L}}=1$	${\left(\frac{\tau_{12}}{S^{L}}\right)}^{2}+\alpha \frac{\sigma_{22}}{Y^{T}}=1\alpha =\frac{Y^{T}}{\left|\sigma_{22}^{Tran}\right|}\left[\left(\frac{\left|\tau_{12}^{Tran}\right|}{S^{L}}\right)-1\right]$

**Table 7:** Comparison with criterion.

Type	E_22_	Relative Error %	Y_T_ (MPa)	Error %	Y_C_(MPa)	Error %
Tsai Hill	9.10	1.56%	117.8	2.33%	254	10.92%
Tsai Wu	9.10	1.56%	80.5	30.07%	268	17.03%
Modified criteria	9.10	1.56%	87.5	23.99%	189	17.47%

**Table 8:** 0 deg specimen dimensions.

Specimen A	Width (mm)	Thickness (mm)	Failure Load (kN)
1	12.1	2.96	43.21
2	12.2	2.94	52.73
3	12.3	2.87	42.46
4	12.5	2.95	47.48

**Table 9:** 90 deg specimen dimensions.

Specimen B	Width (mm)	Thickness (mm)	Failure Load (kN)
1	24.99	2.76	0.872
2	25.21	2.64	0.974
3	25.38	2.59	0.914
4	25.14	2.53	0.987

**Table 10:** 0/90/0/90/0 specimen dimensions.

Specimen C	Width (mm)	Thickness (mm)	Failure Load (kN)
1	24.5	3.0	65.62
2	24.7	2.98	72.65
3	25.4	2.90	75.17

**Table 11:** Comparison between predicted and theoretical values with reference to interface debonding.

Parameter	Longitudinal Strength (MPa)	Predicted Values (MPa)	Error %
E₁₁	150.8	155.2	2.92%
X_T_	2489	2419	2.81%
X_C_	1769	1725	2.49%

**Table 12:** Comparison of theoretical and experimental strength.

Types	Orientations	Theoretical Strength (MPa)	Experimental Strength (MPa)	%Diff.
A	Tensile 0°	1074	893.7	16.78
B	Tensile 0°, 90°, 0°, 90°	642	593.6	7.50
C	Tensile 90°	454	534.3	17.62

**Table 13:** Strength comparison.

Group	Average Strength MPa	Error %	% Variation
*0° 3P specimen*			
0° 3P test	1382.45	--	13.79
RVE	2112.9	13.44	11.12
0° 3P simulation	1829.3	8.7 1	3.2
*+45°, −45° 3P specimen*			
+45°, −45° test	178.5	--	12.40
ABAQUS RVE	208.33	8.6	4.5
+45°, −45° 3P simulation	191.6	11.22	4.9
*(0˚, 90˚, 0˚, 90˚) 3P specimen*			
Laminate test (Experimental)	647.1	11.33	4.55
Laminate simulation	571.8	15.77%	2.45

**Table 14:** Experimental results.

Specimen	Failure Load (kN)	Failure Strength (MPa)	Young’s Modulus E (GPa)
**Category A**			
1	43.21	1326.8	108
2	52.73	1569.1	
3	42.46	1258.09	
4	47.48	1423.6	
**Category B**			
1	0.872	13.70	6.44
2	0.974	15.25	
3	0.914	14.73	
4	0.987	15.39	
**Category C**			
1	65.62	890.1	71.01
2	72.65	985.09	
3	75.17	1019.20	

**Table 15:** Summarized results.

Sample S/No.	Width/mm	Thickness/mm	Area/mm^2^	Max Force (N)	Tensile Strength (MPa)	Modulus (GPa)
1	4.94	3.72	18.38	294.5	16.03	3.920
2	5.50	3.20	17.60	235.5	13.38	4.043
3	4.98	2.28	11.35	134.5	11.85	3.498
4	5.09	2.93	14.91	190.0	12.74	3.916
5	5.09	4.21	21.43	246.5	11.50	5.106
Average values					13.09	4.096
Standard deviation					1.795	0.601
Discrete Coefficient					13.70	14.66

## Data Availability

No repositories involved or shared.
